# Exploring the reporting, intake and recommendations of primary food sources of whole grains globally: a scoping review

**DOI:** 10.1017/S0007114524002678

**Published:** 2024-11-28

**Authors:** Elissa J. Price, Eden M. Barrett, Marijka J. Batterham, Eleanor J. Beck

**Affiliations:** 1 School of Health Sciences, Faculty of Medicine and Health, University of New South Wales, Sydney, NSW, Australia; 2 The George Institute for Global Health, Faculty of Medicine and Health, University of New South Wales, Sydney, NSW, Australia; 3 National Institute for Applied Statistics Research Australia and Statistical Consulting Centre, School of Mathematics and Applied Statistics, Faculty of Engineering and Information Sciences, University of Wollongong, Wollongong, NSW, Australia; 4 School of Medical, Indigenous, and Health Sciences, Faculty of Science, Medicine, and Health, University of Wollongong, Wollongong, NSW, Australia

**Keywords:** Whole grain, Food sources, Whole-grain calculation, Dietary guidelines, Epidemiology

## Abstract

Whole-grain intake is associated with reduced risk of non-communicable diseases. Greater understanding of major food sources of whole grains globally, and how intake has been quantified, is essential to informing accurate strategies aiming to increase consumption and reduce non-communicable disease risk. Therefore, the aim of this review was to identify the primary food sources of whole-grain intake globally and explore how they are quantified and reported within literature, and their recommendation within respective national dietary guidelines. A structured scoping review of published articles and grey literature used a predefined search strategy across electronic databases. Data were extracted and summarised based on identified outcomes (e.g. primary sources of whole-grain intake and quantification methods). Dietary intake values were noted where available. Thirteen records across twenty-four countries identified bread and bread rolls, and ready-to-eat cereals as primary sources of whole-grain intake in Australia, New Zealand, Europe, the UK and Northern America. Elsewhere, sources vary and for large parts of the world (e.g. Africa and Asia), intake data are limited or non-existent. Quantification of whole grain also varied across countries, with some applying different whole-grain food definitions, resulting in a whole-grain intake based on only consumption of select ‘whole-grain’ foods. National dietary guidelines were consistent in promoting whole-grain intake and providing examples of country-specific whole-grain foods. Consistency in whole-grain calculation methods is needed to support accurate and comparative research informing current intake evidence and promotional efforts. National dietary guidelines are consistent in promoting whole-grain intake; however, there is variability in recommendations.

## Graphical abstract

**Figure:**
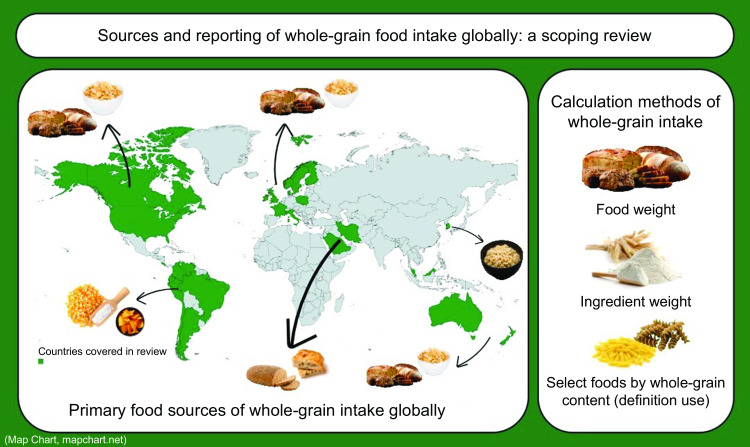

Non-communicable diseases are the leading cause of death and disability worldwide and attribution continues to increase, secondary to population growth and ageing^(1)^. Primary contributors to disease burden include CVD, cancer, chronic respiratory disease and type 2 diabetes^(1)^. Consumption of at least 90 g of whole-grain food per d is associated with reduced risk of these primary contributors and associated conditions, including a 19 % risk reduction for CHD, 22 % for CVD, 15 % for total cancer mortality and 51 % reduced risk for type 2 diabetes^(2)^. Despite the evidence, global whole-grain intake remains well below recommended amounts^(3–6)^. Additionally, interpreting the whole-grain quantity in 90 g of whole-grain food is difficult. Foods contain different amounts of whole grain, for example, a whole-grain bread that aligns with a whole-grain food definition requiring at least 50 % whole-grain content^(7)^ may contain anywhere between 50 and 100 % whole-grain ingredients. Therefore, two slices of whole-grain bread (approximately 60 g whole-grain food) may not contribute the same amount of whole grain to the diet as the amount could vary, for example, from 30 to 60 g whole grain.

Evidence suggests that focusing on intakes of specific foods, such as whole-grain foods, and dietary patterns, rather than individual nutrients, is most relevant to improve cardiometabolic health and reduce non-communicable disease risk^(8)^. Therefore, a greater understanding of major food sources of whole grains around the world is essential to designing and implementing informed strategies to encourage increased whole-grain consumption and concordantly reduce diet-related chronic disease. This includes understanding primary these food sources in both a global context and individual cultural contexts, as well as how they are recommended in guidelines.

Considering the reporting of whole-grain intake to inform promotion of whole grains also requires the methods of measurement of the researcher’s reporting intake. Definitions of whole grains have been relatively consistent throughout literature over time, similar to the global consensus definition of the Whole Grain Initiative (WGI)^(7)^, namely ‘Whole grains shall consist of the intact, ground, cracked, flaked or otherwise processed kernel after the removal of inedible parts such as the hull and husk’. All anatomical components, including the endosperm, germ, and bran must be present in the same relative proportions as in the intact kernel^(7)^. However, a lack of consensus on a definition of a whole-grain food, means that significant variations have been found when exploring whole-grain consumption^(9,10)^. As described, the same weight of a food, even foods high in whole grain, may contain widely different quantities of whole grain. Therefore, for accuracy in reporting whole-grain intakes it is recommended to quantify the amount of whole grain in the food in grams on a dry-weight basis^(11)^. Whole-grain data in this form further allows ease of comparison across different countries’ national intakes. However, often dietary intake of whole grains has been reported based on intake from foods containing whole grain or certain quantities of whole grain^(12,13)^. While consumers may need messages to encourage intake described as foods, ready comparison between reported dietary intakes and intake recommendations is only possible if grams of whole-grain intake are used.

Dietary guidelines worldwide provide dietary-related messaging and advice, enabling informed and healthy food choices for optimal health and well-being of individuals. These guidelines often convey their information in terms of types and amounts of foods, and food groups, such as in the Australian Dietary Guidelines (ADG) or the Eatwell Guide in the UK^(14,15)^. Although the evidence base for dietary intake and health is generally consistent, dietary guidelines are sometimes different due to varied eating patterns and cultural preferences. Considering whole-grain food sources and how whole-grain foods are represented in dietary guidelines is necessary to consider their promotion and its impact on existing low whole-grain intakes globally.

Research has identified that a multi-pronged approach to increase whole-grain intake is essential^(16)^, and understanding foods we eat, how they are quantified and how they are promoted provides a basis for further action. Therefore, the aim of this review was to identify the primary food sources of whole-grain intake globally and explore how they are quantified and reported within literature and their recommendation within respective national dietary guidelines. Intake data were collected where available.

## Methods

A scoping review was selected to explore the extent of both scientific and grey literature on the reporting of primary food sources of whole-grain intake globally, methods of quantification of whole-grain intake and how these foods are considered within respective dietary guidelines. The review process followed a five-stage framework established by Arksey and O’Malley^(17)^. Findings of the study were reported according to the Preferred Reporting Items for Systematic Reviews and Meta-Analyses extension for scoping review (PRISMA-ScR) guidelines^(18)^ (Supplementary Online Material). The study protocol was registered with the Open Science Framework on 16 January 2023 (registration DOI https://doi.org/10.17605/OSF.IO/GJUWD). This review did not require ethics approval.

Electronic bibliographic databases searched included CINHAL plus, MEDLINE, PubMed, Scopus and Web of Science from database inception until March 2024. Search terms were informed by the research question and included free text terms, subject headings and synonyms. Search terms included ‘wholegrain*’, ‘whole grain*’, ‘whole-grain*’, ‘dietary intake’, ‘food intake’, ‘eating patterns’, ‘nutrient intake’ and ‘consumption’. Initially, there were nil limitations on text availability, language, publication date or study design (however, papers were likely to be cross-sectional). This original search strategy was supplemented with a search of reference lists of relevant studies, grey literature including an online search of Google search operators and manual searching of national government websites. Finally, the networks of the Whole Grain Initiative International (WGI) Working Group on Whole Grain Intake Recommendations were contacted to ensure comprehensive identification of all potentially relevant published and unpublished data sources.

To be eligible for this review, papers were required to include data on intakes of adults ≥18 years of age and of any sex. Studies reporting on all ages were still eligible; however, only adult data were extracted for this review. Studies were also required to report whole-grain intake data that is nationally representative of the population, and papers reporting most recent data were prioritised, if countries or region were in duplicate. Where data on whole-grain food sources at the non-nationally representative level, but representing large sample sizes was available, papers were included to inform the discussion of primary food sources of whole-grain intake only and not the reporting of intake amounts. Studies were excluded if analyses focused solely on participants who are <18 years of age and/or are referred to as ‘children’ or ‘adolescents’ or failed to report primary food sources of whole-grain intake, as this was the primary aim of the study.

All relevant citations were collated into EndNote version 20 and exported to Covidence systematic review software (Veritas Health Innovation, Melbourne, Australia, 2020) where removal of duplicates was automatic. To assess citation eligibility, screening involved two researchers (EJP and EJB) applying the eligibility criteria to article titles and abstracts, followed by full-text review. Discrepancies were resolved through discussion. The extracted data included specific details of the citation, country, study design, study population, sample size, whole-grain calculation method, including if a whole-grain food definition was used, and key findings (intake amounts, primary sources of whole-grain intake as reported) which was synthesised in a table to address the review question. Non-nationally representative level studies included to inform food source discussion were listed separately at the end of relevant tables. Data extraction was undertaken by a single researcher (EJP) and continuously reviewed by the research team and discussed if a consensus was required. Whole grains, and primary food sources of whole grains, were then considered in context of their respective national dietary guidelines. National dietary guidelines were primarily identified through the FAO^(19)^, or additional Google searching.

## Results

**Figure 1.**
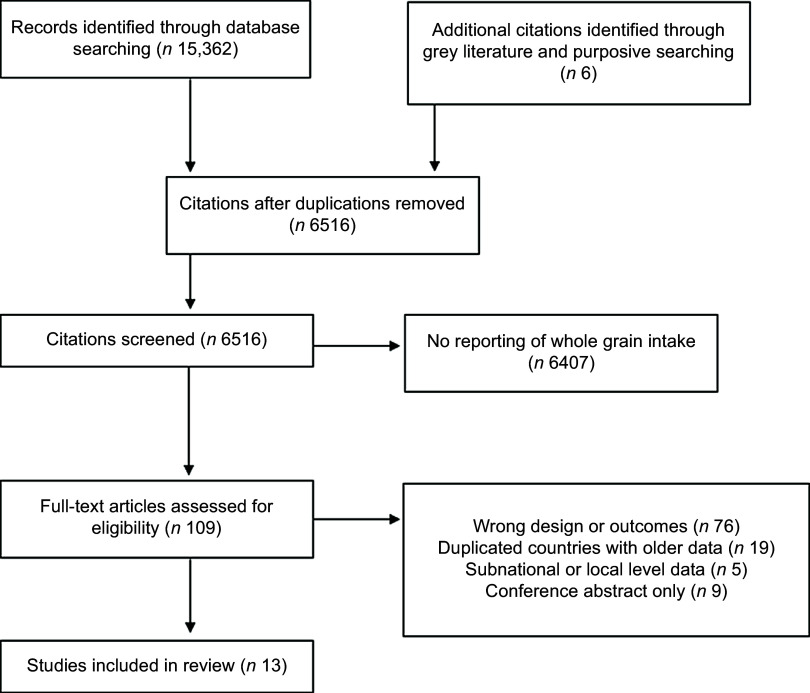
Preferred Reporting Items for Systematic Reviews and Meta-Analyses extension for scoping review (PRISMA-ScR) of included articles relating to national whole-grain intake data (amount and sources of intake).

**Figure 2.**
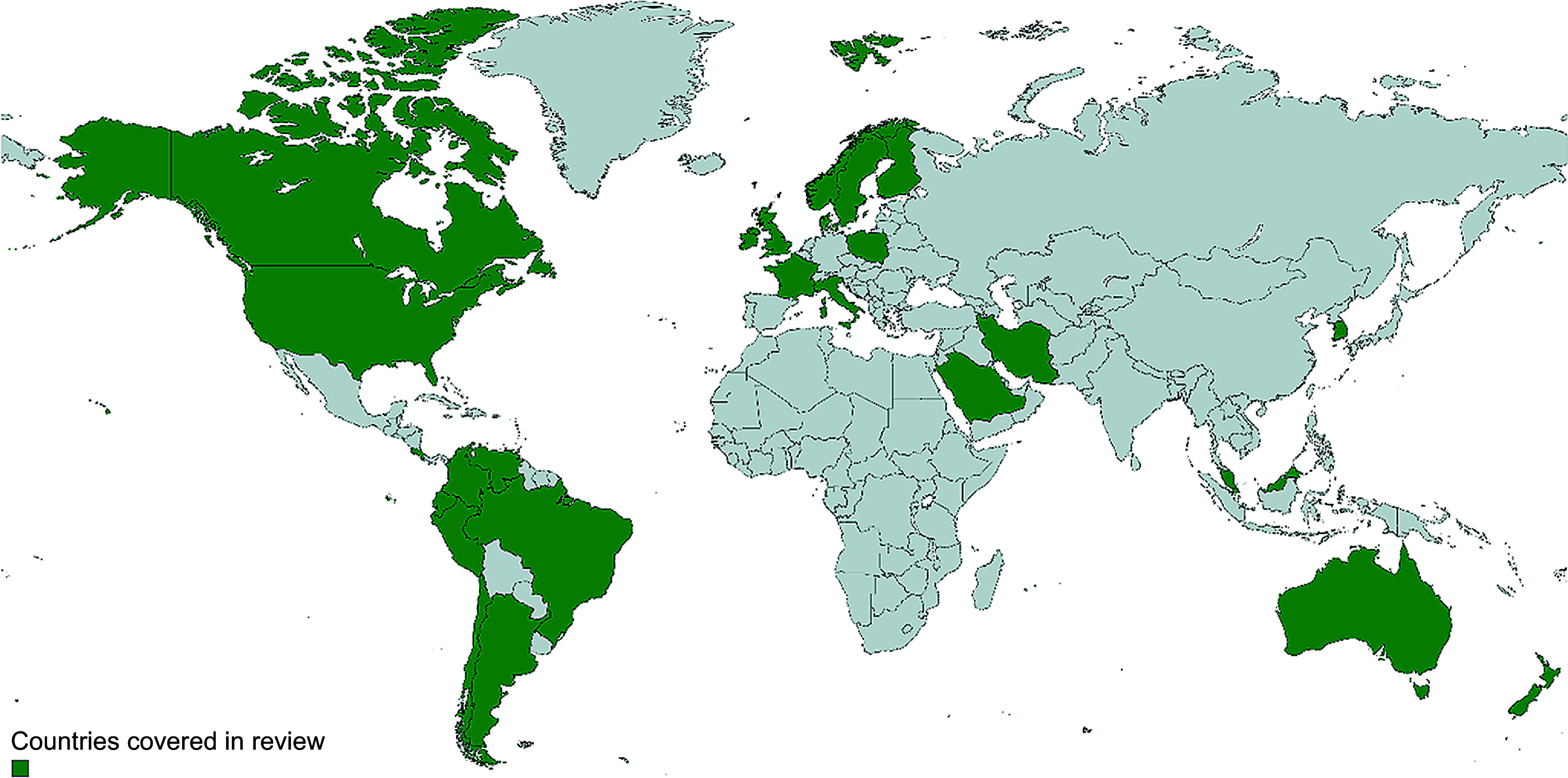
World map representing the geographic coverage of studies included reporting on primary food sources of whole grain. Green areas refer to the location where the included studies were conducted (Map Chart, mapchart.net).

This systematic search identified 6516 records. Following the removal of duplicate papers and application of the eligibility criteria via title and abstract screening, 109 studies required full-text review, with a final ten studies from scientific literature^(3–6,10,12,13,20,21)^ and three items from the grey literature and purposive searching included for synthesis^(22–24)^ (Fig. 1). All thirteen records included data from nationally representative cross-sectional surveys and participants numbers ranged from 706 to 39 775 (Table 1). Countries or regions covered include Argentina, Australia, Brazil, Canada, Chile, Columbia, Costa Rica, Denmark, Ecuador, Finland, France, UK (England, Scotland, Wales and Northern Ireland), Ireland, Italy, South Korea, New Zealand, Norway, Peru, Singapore, Sweden, USA and Venezuela (*n* 24) (Fig. 2). Of the ninety-six records excluded after full-text assessment, nineteen were excluded as they contained duplicate older data, and five due to containing non-nationally representative data. However, although formally excluded, these twenty-four records reported information relevant to inform discussions, allowing greater country or region coverage and exploration of changes in intake over time and thus were set aside for this purpose only. Countries or regions with duplicate older data included Canada, France, UK, Italy, Latin America and USA. Countries reporting whole-grain food sources at the non-national level were Iran, Saudi Arabia, Barbados, Malaysia and Poland^(25–29)^. Few studies were found reporting on whole-grain intakes in Asian countries, and no studies were found for countries in Africa.

**Table 1.** Characteristics of studies included in review reporting primary sources of whole grain intake globally

Citation	Country	Scale	Study design	Study population	Sample size
Bellisle *et al.*, 2014	France	National	Cross-sectional	Comportements et Consommations Alimentaires en France (CCAF) 2009–10.	1389
Cereal Partners Worldwide Nestle and General Mills, 2015	New Zealand	National	Cross-sectional	New Zealand Consumption and Attitudinal Survey (2014) (unpublished)	706
Du *et al.*, 2022	USA	National	Cross-sectional	National Health and Nutrition Examination Survey (2003–18)	39 775
Fisberg *et al.*, 2021	Latin America (Argentina, Brazil, Chile, Columbia, Costa Rica, Ecuador, Peru and Venezuela)	National	Cross-sectional	Latin American Study of Nutrition and Health (ELANS) 2014–15.	9128
Galea *et al.*, 2017	Australia	National	Cross-sectional	National Nutrition and Physical Activity Survey (NNPAS) 2011–12.	12 153
Health Promotion Board, 2010	Singapore	National	Cross-sectional	National Nutrition Survey (NNS) 2010	1627
Kyrø *et al.*, 2012	Scandinavia (Norway, Sweden, and Denmark)	National	Cross-sectional	Subpopulation of the HELGA cohort 1992–98.	8702
Mann *et al.*, 2015	UK (England, Scotland, Wales and Northern Ireland)	National	Cross-sectional	National Diet and Nutrition Survey 2008–11	3073
O’Donovan *et al.*, 2018	Ireland	National	Cross-sectional	2008–10 National Adult Nutrition Survey (NANS)	1500
Ruggiero *et al.*, 2019	Italy	National	Cross-sectional	Italian Nutrition & Health Survey (INHES)	5805
Smith *et al.*, 2021	Canada	National	Cross-sectional	2015 Canadian Community Health Survey (CCHS)	20 487
Tammi *et al.*, 2021	Finland	National	Cross-sectional	FinRavinto 2017 Survey	1655
Seungmin, 2011	South Korea	National	Cross-sectional	The Korean National Health and Nutrition Examination Survey 2007–08	8836

There were several important summary findings in this review. First, primary food sources and quantities of whole-grain intake were relatively consistent across the Western world (Australia, New Zealand, Europe, the UK and Northern America^(3–6,10,13,21,22,30)^ but differed in other regions^(12,20)^. Second, the methods of calculation of whole-grain intake varied considerably between studies and therefore countries. Additional further variation is also likely due to application of different whole-grain food definitions used to calculate intake present in some studies. Finally, recommendations regarding whole-grain intake were similar throughout national dietary guidelines and were tailored to respective primary food sources.

The primary source of whole-grain intake in Australia, Denmark, France, Ireland, New Zealand, Norway, Singapore and the UK are bread and bread rolls, followed by ready-to-eat cereals or other cereals (Table 2)^(3–6,20,22,30)^. Canada and Sweden reported these same primary sources; however, ready-to-eat cereals or other cereals preceded bread and bread rolls^(3,13)^. Ready-to-eat cereals or other cereals were also the primary source for the USA; however, these were followed by cooked grains and cereals, savoury snacks/crackers, and then bread^(10)^. Bread was the primary food source in Italy; however, it was followed by whole-grain biscuits, whole-grain pasta and then whole-grain breakfast cereals^(21)^. Finland specified rye bread as the primary food source, and porridges, mixed breads, and breakfast cereal products followed in that order^(23)^. Within Argentina and Chile, commercial whole wheat bread was reported as the primary source of whole-grain intake, but there was variation among other countries in Latin America^(12)^. Examples of other primary sources across Latin America include corn chips, caramel or sugar-coated popcorn, masa harina corn flour, regular or quick oatmeal, fortified tortilla maize, dry quinoa and regular wheat crackers. South Korea’s primary whole-grain food source was mixed multigrain rice, followed by maize and brown rice for younger males and females^(20)^. For females over ≥40 years of age, the primary food source was maize, followed by mixed multigrain rice and brown rice^(20)^. Four papers exploring primary whole-grain food sources at the non-national level were also identified in this review to inform discussions. These studies reported on intakes in Iran^(25)^, Saudi Arabia^(26)^, Barbados^(27)^, Malaysia^(28)^ and Poland^(29)^ and interestingly identified similar primary whole-grain food sources such as oatmeal and breads, including oat bran, whole wheat and multigrain varieties. In addition to these, brown rice, and Barbari, Taftoon, and Sangak breads were reported as primary food sources of whole grain in Malaysia and Iran, respectively^(25,28)^.

**Table 2.** Intakes and primary food sources of whole grains globally and recommendations in the context of respective definitions and national dietary guidelines

Citation	Country	Total intake amounts	Primary food sources of whole grain as reported	Dietary guideline recommendations
Bellisle *et al.*, 2014	France	4·7 g/d (total pop); 14·4 g/d (consumers)	Breads and toasts, ready-to-eat cereals, pastas, rice, and cooked cereals and cereal bars	‘Increase the consumption of starchy foods, including cereals (especially whole grain cereals, which provide fibre), potatoes, pulses, etc. They should be present at each meal … Eat every day and give preference to whole grain and minimally processed products’.^(31)^
Cereal Partners Worldwide Nestle and General Mills, 2015	New Zealand	Not stated	Bread, hot cereal, ready-to-eat cereal, muesli and rice	‘Enjoy a variety of nutritious foods every day including: … Grain foods, mostly whole grain and those naturally high in fibre’.^(32)^
Du *et al.*, 2022	USA	0·81-ounce equivalents/2000 kcal/d	Ready-to-eat cereals, cooked grains and cereals, savoury snacks/crackers and breads	‘Three or more-ounce equivalents of whole grain per day … Healthy dietary patterns include whole grains and limit the intake of refined grains. At least half of total grains should be whole grains’.^(33)^
		0·53-ounce equivalents/2000 kcal/d	Ready-to-eat cereals, cooked grains and cereals, savoury snacks/crackers and breads	
		1·05-ounce equivalents/2000 kcal/d	Ready-to-eat cereals, breads, cooked grains and cereals and savoury snacks/crackers	
		0·73-ounce equivalents/2000 kcal/d	Ready-to-eat cereals, cooked grains and cereals, savoury snacks/crackers and breads	
		0·95-ounce equivalents/2000 kcal/d	Ready-to-eat cereals, cooked grains and cereals, breads and savoury snacks/crackers	
Fisberg *et al.*, 2021	Argentina	14·4 g/d	Commercial whole wheat bread and homemade or bakery whole wheat bread	‘Eat legumes; cereals, preferably wholemeal; potato; sweet potato; corn or cassava’.^(34)^
		11·1 g/d	Commercial whole wheat bread and homemade or bakery whole wheat bread	
	Brazil	10·6 g/d	Crackers, wheat, regular and corn chips, ingredient fat not known	‘White rice and white wheat flour have lower amounts of dietary fibre and micronutrients, which are lost in the refining process. Less processed versions of these foods, such as brown rice and whole-wheat flour, are preferable’.^(35)^
		8·2 g/d	Corn chips and popcorn from package, regular	
	Chile	11·8 g/d	Commercial whole wheat bread and caramel or sugar-coated popcorn	‘Frequent consumption of fruits is recommended, vegetables, whole grains, nuts, legumes, water, milk and its derivatives, as well as marine products high in omega 3’.^(36)^
		8·9 g/d	Commercial whole wheat bread and caramel or sugar-coated popcorn	
	Colombia	19·6 g/d	Masa harina corn flour and steel cut dry oatmeal	NA
		17·4 g/d	Masa harina corn flour and steel cut dry oatmeal	
	Costa Rica	20·8 g/d	Regular or quick oatmeal and fortified tortilla maize	‘The basis of the daily diet should be cereals, legumes, and vegetables’.^(37)^
		18·9 g/d	Regular or quick oatmeal and fortified tortilla maize	
	Ecuador	14·3 g/d	Regular or quick oatmeal, whole wheat bread rolls, cookies, homemade or bakery whole wheat bread	‘Let’s eat better by combining legumes with cereals like rice, maize or quinoa’.^(38)^
		12·4 g/d	Regular or quick oatmeal and whole wheat bread rolls	
	Peru	7·1 g/d	Dry, regular, or quick oatmeal and dry quinoa	‘Take care of your weight by consuming rice, pasta and bread in moderation … Ultra-processed food examples: sweetened breakfast cereals and cereal bars’.^(39)^
		13·6 g/d	Dry, regular, or quick oatmeal and dry quinoa	
	Venezuela	13·6 g/d	Flour, maize, masa harina and flour, white whole wheat	‘To obtain a varied diet, foods must be consumed daily of the three food groups … Grains, cereals, tubers and bananas’.^(40)^
		14·6 g/d	Flour, maize, masa harina and flour, white whole wheat	
Galea *et al.*, 2017	Australia	21·2 g/d (total pop); 38·4 g/d (consumers)	Regular breads and bread rolls and ready-to-eat cereals	‘Enjoy a wide variety of nutritious foods from these five groups every day: Grain (cereal) foods, mostly wholegrain and/or high cereal fibre varieties, such as breads, cereals, rice, pasta, noodles, polenta, couscous, oats, quinoa and barley’.^(14)^
Health Promotion Board, 2010	Singapore	27 % of adults consumed at least one serving of WGF daily	Wholemeal bread and whole-grain cereals, brown rice/porridge and whole-grain noodles	‘5–7 servings of rice and alternatives (of which at least one serving should be a wholegrain product)’.^(41)^ ‘Fill a quarter of your plate with whole grains’.^(42)^
Kyrø *et al.*, 2012	Norway	44 g/d	Whole-grain bread, breakfast cereals and other products	‘It is recommended to have an intake of at least 90 g/d of whole grains (including whole grains in products), with likely further benefits of higher intakes. Whole grain cereals other than rice should preferentially be used’.^(43)^
	Sweden	F 35 g/d; M 49 g/d	Breakfast cereals, whole-grain bread, and whole-grain crispbread and rusks	As above.
	Denmark	F 31 g/d; M 41 g/d	Whole-grain bread, breakfast cereal and other wholegrain products	As above.
Mann *et al.*, 2015	UK (England, Scotland, Wales and Northern Ireland)	26·7 g/10 MJ/d 26·6 g/10 MJ/d 14·7 g/10 MJ/d 45 % adults consumed ≥1 serve/d	Whole-grain breads and ready-to-eat cereals	‘Starchy food should make up just over a third of the food we eat. Choose higher fibre or wholegrain varieties, such as wholewheat pasta and brown rice, or simply leave the skins on potatoes’.^(15)^
O’Donovan *et al.*, 2018	Ireland	27·8 g/d (total pop); 30·6 g/d (consumers)	Bread and bread rolls, ready-to-eat cereals, and other breakfast cereals, for example, porridge	‘Choose wholemeal and wholegrain breads, cereals, pasta, and brown rice … 3–5 servings/d of wholemeal cereals and breads, potatoes, pasta and rice … Wholemeal and wholegrain cereals are best … Enjoy at each meal’.^(44)^
Ruggiero *et al.*, 2019	Italy	55·3 % nil; 17·5 % <1 x/week; 27·2 % ≥1 x/week	Whole-grain bread, whole-grain biscuits, whole-grain pasta, whole-grain breakfast cereals and whole-grain soups	‘Regularly consume bread, pasta, rice, and other grains (preferably wholegrain), avoiding too much fat condiments … At least half of the cereal portions consumed daily should be wholegrain’.^(45)^
Smith *et al.*, 2021	Canada	28·9 g/d to 219·9 g/d	Whole-grain oat and high-fibre breakfast cereal and whole-grain and whole wheat bread	‘Enjoy a variety of whole grain foods every day, such as: quinoa, whole grain pasta, whole grain bread, whole oats or oatmeal, whole grain brown or wild rice’.^(46)^
Tammi *et al.*, 2021	Finland	F 47 g/d; M 63 g/d	Rye bread, porridges, mixed breads, breakfast cereal products, pastries, buns, cookies, pasta and rice, wheat breads and other	‘It is recommended to have an intake of at least 90 g/d of whole grains (including whole grains in products), with likely further benefits of higher intakes. Whole grain cereals other than rice should preferentially be used’.^(43)^
Seungmin, 2011	South Korea	F 58·9 % nil, 26·6 % <20 g/d, 14·6 % >20 g/d; M 61·1 % nil, 23·2 % <20 g/d, 15·7 % >20 g/d	M ≥ 20 years and F < 40 years: boiled mixed multigrain rice, maize and brown rice. F ≥ 40 years: maize, mixed multigrain rice and brown rice	‘Eat a variety of foods including rice and other grains, vegetables, fruits, milk and dairy products, meat, fish, eggs, and beans … Grains 2–4 servings/d’.^(47)^
Białek-Dratwa *et al.*, 2023	Silesia Region (Poland)	NA^*^	Wholemeal bread, oatmeal, buckwheat groats, wholemeal pasta, brown rice, muesli and bran	‘Eat more whole-grain cereal products (e.g. oatmeal, wholemeal bread, wholemeal pasta, groats)’.^(48)^
Esmaillzadeh *et al.*, 2004	Tehran (Iran)	93 ± 29 g/d (F 90 ± 24 g/d; M 98 ± 36)^*^	Barbari, Taftoon and Sangak breads	‘When choosing bread and cereals, it is better to choose whole-grain (i.e. with bran not removed) types as far as possible’.^(49)^
Jozaa Zaidan Al, 2016	Saudi Arabia	57·3 % nil, 25·3 % <half grains as WG, 11·3 % consumed half as WG, 6·1 % >half grains as WG^*^	Bread, bran bread, crushed grain bread and bran biscuits	‘Choose grain such as rice and grain products such as bread prepared with little or no added sugar, fat or salts … Select food prepared from whole grains or cereals. Choose brown bread better than white bread made from processed cereals’.^(50)^
Sharma *et al.*, 2008	Bardbados	Not reported^*^	Oat bran bread, multigrain bread and whole wheat bread	‘Whole grain bread, cereals, and pasta are high fibre choices’.^(51)^
Subramanian *et al.*, 2019	Malaysia	13 % daily consumers, 26 % >3 x/month, 23·3 % >3 x/week, 22 % <3 x/week, and 15·6 % <3 x/month.^*^	Oatmeal, wholegrain and wholemeal bread, brown rice, biscuits or wholegrain enriched bars	‘Eat 3–5 servings of cereals, cereal-based products and tubers daily according to your energy needs and physical activity level … Choose at least half of your cereals and cereal-based products from whole grains’.^(52)^

WG, whole grain.

* Whole-grain intake data not reported of nationally representative population.

Calculation of whole-grain intake values varied considerably across countries, and in some cases, different whole-grain food definitions were applied, resulting in a whole-grain intake based on only consumption of select ‘whole-grain’ foods (Table 3). Methods of reporting population mean whole-grain intake therefore varied secondary to these differences in whole-grain calculation and whole-grain food definitions used (Table 3). Seven studies (twelve countries) included in this review calculated whole-grain intake by including quantities of whole grain from any food containing a whole-grain ingredient regardless of the quantity of whole grain contained. This was the case for Australia, Denmark, Finland, France, Ireland, South Korea, Norway, Sweden and the UK^(3–6,20,23,30)^. These same seven studies (twelve countries) calculated whole grain as grams of whole-grain ingredient per d. The UK further provided calculations applying various definitions to identify a whole-grain food. That is, not all grams of whole-grain intake would be included in these values, only grams of whole grain from foods meeting those definitions. Definitions included foods containing ≥10 % of whole grain and ≥51 % of whole grain. Most of these studies reported population mean whole-grain intake as grams per d, some providing this value for both whole-grain consumers and the total population, or by sex. Unlike the others, however, South Korea reported the percentage of the population meeting predefined cut-offs including nil intake, <20 g/d and >20 g/d for both males and females. All other studies calculated intake using varied definitions and then reported as grams from select foods, or in some other measurement such as serves.

**Table 3.** Whole-grain calculation and reporting methods of studies reporting primary sources of whole-grain intake globally included in review

Citation	Country	Sample size	Whole-grain calculation	Whole-grain food definition
Bellisle *et al.*, 2014	France	1389	Grams WG/d	Any food containing whole grain regardless of amount
Cereal Partners Worldwide Nestle and General Mills, 2015	New Zealand	706	Serves WGF/d	Core grain foods containing whole grain ingredients
Du *et al.*, 2022	USA	39 775	Oz. eq./2000 kcal (grams WGF/2000 kcal/d)	≥50 % of the grain- or flour-containing component as whole grain ingredients (DGA) ≥51 % of the RACC food weight as whole grain ingredients (FDA) Grain-rich foods having ≥1·1 g of fibre per 10 g of carbohydrates (AHA) ≥8 g of whole grain per 30 g of the product (AACCI) ≥8 g of whole grain ingredients per labelled serving (WGC)
Fisberg *et al.*, 2021	Latin America (Argentina, Brazil, Chile, Columbia, Costa Rica, Ecuador, Peru and Venezuela)	9128	Grams WGF/d	Any food containing whole grain regardless of amount Foods containing ≥ 50 % whole grain
Galea *et al.*, 2017	Australia	12 153	Grams WG/d	Any food containing whole grain regardless of amount
Health Promotion Board, 2010	Singapore	1627	Grams WGF/d	Not stated
Kyrø *et al.*, 2012	Scandinavia (Norway, Sweden, Denmark)	8702	Grams WG/d	Any food containing whole grain regardless of amount
Mann *et al.*, 2015	UK (England, Scotland, Wales and Northern Ireland)	3073	Grams WG/d and serves WG/d (16 g = 1)	Any food containing whole grain regardless of amount All foods containing ≥10 % whole grain All foods containing ≥51 % whole grain
O’Donovan *et al.*, 2018	Ireland	1500	Grams WG/d	Any food containing whole grain regardless of amount
Ruggiero *et al.*, 2019	Italy	5805	Freq. WGF/week	Not stated
Smith *et al.*, 2021	Canada	20 487	Grams WGF/d	Canada’s food guide ‘Grain products – whole’; excludes mixed dishes
Tammi *et al.*, 2021	Finland	1655	Grams WG/d	Any food containing whole grain regardless of amount
Seungmin, 2011	South Korea	8836	Grams WG/d	Any food containing whole grain regardless of amount

A single study reporting intakes for Argentina, Brazil, Chile, Columbia, Costa Rica, Ecuador, Peru and Venezuela calculated whole grain as grams of whole-grain food per d and reported mean population intakes using the same^(12)^. This study considered varying definitions of whole-grain foods when calculating quantities of whole-grain intake, including calculations for foods containing any whole grain amount (as per Australia, Sweden, Denmark, etc above) and then only for foods containing ≥50 % whole-grain ingredients^(12)^.

The article reporting on national whole-grain intakes in New Zealand defined a whole-grain food as core grain foods containing whole-grain ingredients and did not consider whole-grain quantities from discretionary foods. The same article also reported whole grain as serves of whole-grain food per d with nil specificity of whole-grain quantity in a single serve^(22)^. Mean population intakes were not reported for New Zealand, rather population percentages of intake frequency were reported. Similarly, the study from Canada defined a whole-grain food according to the Canada Food Guide ‘Grain products – whole’ which excluded calculation of whole-grain quantities in products not considered ‘whole’ and mixed dishes^(13)^. Further, whole grain was calculated as grams of whole-grain food per d, and population intake was provided as a range using this metric. The study from Singapore also calculated whole grain as grams of whole-grain food per d but did not state how they defined a whole-grain food^(24)^. Population intake amounts were reported as percentage of adults consuming at least one serving of whole-grain food per d. Likewise, Italian data did not state how a whole-grain food was defined and calculated whole grain as frequency of whole-grain food intake per week, thus giving no indication of quantities of intake^(21)^. Amounts of intake were reported as the percentage of the population consuming no whole grain, <1 x/week and ≥1 x/week.

The study reporting intakes for the USA calculated and reported whole-grain intake as ounce equivalents of a whole-grain food per 2000 kcal/d and provided no further indication of whole-grain quantity in a single ounce equivalent^(10)^. Whole-grain foods were defined as foods that met certain whole-grain content definitions outlined by the Dietary Guidelines for Americans (DGA), Food and Drug Administration (FDA), American Heart Association AHA), American Association of Cereal Chemists International (AACCI) and the Whole Grain Council (WGC)^(10)^. These respective definitions included foods containing ≥50 % of the total grain weight as whole-grain ingredients, food containing 51 % or more of the reference amount customarily consumed (RACC) food weight as whole-grain ingredients, grain-rich foods having ≥1·1 g of fibre per 10 g of carbohydrates, foods with ≥8 g of whole grains per 30 g of the product and foods containing ≥8 g of whole-grain ingredients per labelled serving^(10)^. There was no calculation completed without application of any definition.

Almost all dietary guidelines suggest that a majority, or more than half, of grain intake should be that of whole grain or wholemeal varieties or recommend swapping to these types. However, of the countries included in this study, four national dietary guidelines provided more specific quantitative whole-grain intake recommendations. The Healthy Ireland Food Pyramid recommends 3–5 servings/d of ‘wholemeal cereals and breads, potatoes, pasta, and rice’ food group and further says ‘Wholemeal and wholegrain cereals are best. Enjoy at each meal’^(44)^. The Nordic Nutrition Recommendations state that ‘it is recommended to have an intake of at least 90 g/d of whole grains (including whole grains in products), with likely further benefits of higher intakes’^(43)^. The DGA suggest consumption of three-ounce equivalents of whole grain per d^(33)^. The Singapore food-based Dietary Guidelines recommends an intake of 5–7 servings of rice and alternatives daily (of which at least one serving should be a whole-grain product)^(41)^. Additionally, the General Dietary Guidelines for Koreans recommends 2–4 servings/d of grains; however, it does not specify whole-grain varieties^(47)^. Seven dietary guidelines specifically include whole-grain bread in their recommendations and eight specifically mention whole-grain cereals or breakfast cereals in recommendations. Dietary guidelines in the Latin America region differ in their food sources mentioned, such as the dietary guidelines for the Argentinian population stating, ‘eat legumes; cereals, preferably wholemeal; potato; sweet potato; corn or cassava’ and Brazil having no mention of any food groups^(34,35)^.

## Discussion

Whole-grain intake is associated with reduced risk of chronic disease yet identifying the primary food sources of whole-grain intake globally and how they are recommended, to optimise intake, is complicated by the methods and reporting. This scoping review addressed this knowledge gap by synthesising peer-reviewed and grey literature publications to summarise country-specific literature on food sources and methods of calculating intake, as well as how whole-grain foods are promoted within respective national dietary guidelines. This review included thirteen publications, which covered twenty-four countries, and identified that comparison of analyses and reporting of global whole-grain intakes is limited by inconsistencies in calculation of whole-grain quantities. Regardless of the methods and reporting, however, whole-grain foods are consistently recommended throughout national dietary guidelines.

The current study reported major food sources of whole-grain intake as they were reported in the studies included in this review and identified breads and ready-to-eat cereals as the primary food sources of whole-grain intake within Australia, New Zealand, Singapore, Europe, Northern America and the UK. Whilst this provides insight to major sources in respective countries, each have different food sources, and group foods differently, which may also impact the reporting of major sources. For example, data from Australia considered food groups such as ‘regular bread and bread rolls’ and ‘ready-to-eat cereals’^(5)^, whereas data in New Zealand detailed categories of breakfast-type cereal intake, including ‘hot cereal’, ‘ready-to-eat cereal’ and ‘muesli’^(22)^(Table 2). Although this makes direct comparisons difficult, we could infer that grouping of breakfast cereals in New Zealand (hot and cold) under a single category may show that they contribute more to whole-grain intake than bread, which was the highest contributing source listed. Similarly for Sweden, breakfast cereals were the major whole-grain source, in contrast to Norway and Denmark, where soft whole-grain breads were the major source. However, in Sweden, whole-grain bread was split across two categories: bread, and crispbread and rusks, and if combined, bread becomes the major source of whole grains overall. Sources of whole-grain intake were more varied Latin America and South Korea, including maize-based products, oatmeal, other cooked grains and rice.

Different methods of calculating major food sources may also limit cross-country comparisons. For example, methods included percentage of contribution to whole-grain intake as reported for France^(4)^. Others included percentage of contribution to total energy intake (e.g. Latin America)^(12)^, and percentage ratio of whole-grain *v*. refined-grain to total grain intake for each food group as per the USA^(10)^. Uniform reporting regarding both food groups and calculation methods is therefore a recommendation for future research.

Differences in primary sources of whole-grain food intake in some regions is not surprising. Environmental drivers of food choices and diet-related behaviours are well documented throughout literature and include the like of food availability, cultural practices, social relations, price, time, education and advertising^(53)^. The USA is the largest producer of maize globally, though this does not mean that there is corresponding widespread availability of whole-grain maize products; it is however still a major staple food in Latin America. Despite low overall whole-grain intakes for this region, for those consuming whole grains, maize is a key source which could be in part because of its close availability^(54)^. It is also not surprising that many regions or countries consume whole grains as products like breads and cereals due to their familiarity and convenience. Research exploring whole-grain intake in Nordic countries and the St. Petersburg region identified the time-saving effect of consuming convenience foods as a common driver of consumption^(55)^. Amounts of total whole-grain intake in certain countries may also result in more varied sources of whole-grain foods reported. For example, in some parts of Latin America where median intake amounts are negligible, more obscure sources of whole grain may be reported, such as caramel or sugar-coated popcorn^(12)^. Future research is required that more closely considers determinants impacting selection of whole-grain foods and how this relates to overall intake, particularly in the context of low whole-grain intakes.

Dietary guidelines are evidence-based messaging tools to support populations and consumers in making healthy dietary choices and habits. The majority of dietary guidelines of regions and countries included in this review promoted whole-grain intake as part of a balanced diet; however, globally only 44 % of countries with established food guides specifically recommend consumption of whole grains^(56)^. Similarly, a majority also recommended that ‘most’ or ‘more than half’ of grain intake be that of whole grains, which is consistent across many food guides emphasising whole grains within a more general grain food statement^(56)^. Research exploring the impacts of a quantitative *v*. qualitative (i.e. descriptive) recommendation on whole-grain intakes, and therefore health, would be insightful. For some countries in the current study, whole-grain intakes where there is a corresponding quantitative recommendation appear to have marginally higher intakes than those with general encouragement of consumption (e.g. 44 g WG/d in Norway *v*. 38·4 g WG/d in Australia, respectively). As national dietary guidelines are often tailored to country-specific intake trends and cultural or religious beliefs, it is not surprising that primary food sources of whole grains listed in this review were often mentioned within respective country dietary guideline recommendations as examples of whole-grain foods to include as part of a healthy diet.

This review found that various methods to calculate whole-grain intake were used when analysing national whole-grain intakes and that often definitions were also applied to identify a whole-grain food with ramifications on the calculation of intake. There was a relatively even split of countries calculating whole grain for any food containing whole grain *v*. only calculating foods containing a specific whole-grain amount (e.g. foods containing ≥10 % whole grain or foods ≥50 % whole grain). As a result, foods with whole-grain content less than these values were not considered in the reporting of national intakes. Some studies also calculated whole grain based on the grams, serves or ounce equivalents of a whole-grain food consumed per d but did not provide further indication of whole-grain quantities in the grams, serves or ounce equivalents of these foods. To exemplify this, a 30 g or 1 oz equivalent serve of a whole-grain food may contain approximately 16 g of whole grain (e.g. bread) or 30 g of whole grain (e.g. dry oats in muesli) or any range of whole grain depending on moisture, percentage whole grain and other ingredients. Inconsistencies in calculation of whole grain when reporting national intakes hinders accurate reporting and limits the ability for cross-country or global comparisons of intake when designing targeted and specified health promotion efforts.

Identification of primary sources of whole-grain intake is also impacted when definitions are used. Of the studies included, one considered multiple definitions in its analyses of whole-grain intake in Latin America and found this impacted the reporting of primary food sources of whole-grain intake in Brazil^(12)^. This was evident when comparing any foods containing whole grain and ≥50 % whole-grain containing foods. Similar findings were evident in the US population when using whole-grain food definitions by the DGA, FDA, AACCI and WGC^(10)^. Similarly, use of varying cut-offs of whole-grain content also changed the primary food sources reported in the UK study^(30)^. Differences in identification of primary food sources of whole grain found in these studies may have unintended impacts on policy direction, such as guidance in the fortification of popular grain foods, where primary sources may be missed due to inaccuracy or inconsistency in reporting. Varying definition use may also affect consumer education and understanding of whole-grain foods. For example, the WGC permits the presence of a Basic Whole Grain Stamp on front-of-pack labelling if it contains ≥8 g of whole-grain ingredients per labelled serving; however, this likely misses some whole-grain foods that may be known to the consumer, causing further confusion.

Whilst this review provides an overview on global differences in calculating and reporting of whole-grain intakes, previous research has looked more closely at impacts of this when assessing associations with health outcomes. Kissock and colleagues concluded that reporting of population whole-grain intake amounts was substantially impacted by the application of a whole-grain food definition in the Australian and Swedish populations, and therefore associations with health outcomes were mildly impacted^(57)^. This is likely because small amounts of whole grain may be in less healthful food choices, limiting their health effects. Therefore, using a definition in calculation of intake may be most relevant in public health promotion with some smaller impacts when measuring health associations^(57)^.

One example of a public health promotion strategy where use of a definition may be pertinent is for direction with front-of-pack labelling. In 2021, the WGI developed the global consensus definition of a whole-grain food which states ‘a whole-grain food shall contain at least 50 % whole-grain ingredients based on dry weight’ and is endorsed by the Cereals and Grains Association, HealthGrain Forum, and AACCI^(7)^. If application of a whole-grain food definition is needed for labelling requirements, to promote or aid consumers in selecting healthier whole-grain products, use of this definition would ideally be adopted. This way, only high whole-grain foods would be encouraged whilst simultaneously limiting consumer scepticism of such labelling, of which has been highlighted in consumer studies^(58)^.

Whole-grain consumption data is missing for many countries and regions, and in some cases, this is due to limited data on the whole-grain composition of foods. Regions with missing data primarily include Africa and Asia, and missing whole-grain food composition data in these regions is likely due to varied definitions of whole grains and whole-grain foods used. For example, in China, for some time analyses of grain intake referenced ‘coarse grains’ to include whole grains but also pulses and bran, and thus substitution or comparison to other national datasets has not previously been fit for purpose. Recent research looking at time trends of whole-grain intake with associations of cancer in China defined a whole-grain food as containing greater than 1 g of dietary fibre per 100 g in respective food composition tables^(59)^. The low-fibre threshold in this definition, however, introduces issues as the fibre content of white rice is 1 g/100 g and white bread typically has 3 g fibre/100 g; therefore, it is considered whole grain without containing whole grains. Others have identified types of whole grain more explicitly as intact or ‘cracked’ whole grain and whole-grain porridges, or wheat products made from flour containing this grain^(60)^. Using available data, rice and wheat are reported as the major sources of grain consumption in China and are often consumed as refined varieties^(61)^. Efforts to encourage consumption of brown rice as a whole-grain source in the Chinese population have been widely unfavourable due to high prices, unique taste and different cooking requirements^(62)^. Whole-grain maize product consumption is characteristic of a traditional dietary pattern in Ethiopia^(63)^. Major sources of grain intake in Africa more generally includes sorghum, pearl millet, fonio, teff and finger millet which are likely to be whole grain. However, definitive classification is difficult without a formal whole grain definition^(64)^. Therefore, a definition for whole grain is needed in this context as well as addition of whole grains to food composition tables^(65)^.

Synthesis of both grey and scientific literature is a strength of this review as it provides a comprehensive summary of primary food sources of whole-grain intake, including how they are reported in literature and recommended in dietary guidelines. However, the review is not without limitations. Due to a lack of data on reporting primary sources of whole-grain intake globally, not all countries or regions are covered in this review as previously discussed. A significant knowledge gap on whole-grain consumption data in densely populated parts of the world is evident, and although we aimed to translate documents on all national data, some may not be included as searches were completed in English language databases, but it is most likely the data do not exist. The cross-sectional design of all studies also introduces limitations inherent in their design regarding examining dietary intake at a singular point in time and thus are unable to infer usual intakes. Lastly, use of different whole-grain food definitions by the studies in this review limits the current findings of primary sources of whole-grain intake.

This review highlights the similarities and differences in sources of whole grain across countries and that inconsistencies exist in the calculation and reporting of primary food sources of whole-grain intake globally. Consistency in whole-grain calculation methods and definitions used to identify whole-grain foods, when necessary, is needed to support accurate and comparative research informing current intake evidence and promotional efforts. National dietary guidelines are consistent in the messaging and promotion of whole-grain intake; however, variation exists as to how they are recommended. Future research needs to be consistent in the calculation and reporting of whole grains and should consider impacts of other dietary messaging models on promoting primary whole-grain food sources.

## Supporting information

Price et al. supplementary material Price et al. supplementary material

## Supplementary material

For supplementary material/s referred to in this article, please visit https://doi.org/10.1017/S0007114524002678
